# Long-term Consequences of Traumatic Brain Injury in Bone Metabolism

**DOI:** 10.3389/fneur.2018.00115

**Published:** 2018-03-05

**Authors:** Nikita M. Bajwa, Chandrasekhar Kesavan, Subburaman Mohan

**Affiliations:** ^1^Musculoskeletal Disease Center, VA Loma Linda Healthcare System, Loma Linda, CA, United States; ^2^Department of Medicine, Loma Linda University, Loma Linda, CA, United States; ^3^Department of Orthopedic Surgery, Loma Linda University, Loma Linda, CA, United States

**Keywords:** osteoporosis, growth hormone, bone formation, bone resorption, heterotopic ossification, fracture repair, neuropeptides

## Abstract

Traumatic brain injury (TBI) leads to long-term cognitive, behavioral, affective deficits, and increase neurodegenerative diseases. It is only in recent years that there is growing awareness that TBI even in its milder form poses long-term health consequences to not only the brain but to other organ systems. Also, the concept that hormonal signals and neural circuits that originate in the hypothalamus play key roles in regulating skeletal system is gaining recognition based on recent mouse genetic studies. Accordingly, many TBI patients have also presented with hormonal dysfunction, increased skeletal fragility, and increased risk of skeletal diseases. Research from animal models suggests that TBI may exacerbate the activation and inactivation of molecular pathways leading to changes in both osteogenesis and bone destruction. TBI has also been found to induce the formation of heterotopic ossification and increased callus formation at sites of muscle or fracture injury through increased vascularization and activation of systemic factors. Recent studies also suggest that the disruption of endocrine factors and neuropeptides caused by TBI may induce adverse skeletal effects. This review will discuss the long-term consequences of TBI on the skeletal system and TBI-induced signaling pathways that contribute to the formation of ectopic bone, altered fracture healing, and reduced bone mass.

## Introduction

Traumatic brain injury (TBI) is the disruption of brain activity due to an external force or violent blow to the head. TBI can lead to a series of physical, cognitive, social, emotional, and behavioral impairments (1) and is the leading cause of death and disability in both combat and civilian populations. More than 1.7 million people in the U.S. experience a TBI annually (1), and it is a major cause of death and disability worldwide, especially in children and young adults. Significant proportions of survivors require hospital care, extended rehabilitation, and may have long-term physical, cognitive, and psychological disorders. Many statistics do not account for individuals who have not reported an injury or received medical care, and disabilities may be significantly higher, often with long-term consequences. TBI may be classified based on severity as mild, moderate, or severe and location of injury and time of lost consciousness (2). Symptoms can range from mild concussions, with symptoms lasting from seconds, to more severe injuries with symptoms lasting years or even death. Individuals who have suffered mild TBI, or concussions, report adverse effects resulting from the TBI(s) months later (3) due to the rotational stress caused by head movement. In fact, TBI is the beginning of an ongoing, possibly lifelong, process that impacts multiple organ systems and bone that is proximal or distal to the site of injury. This review will discuss the consequences that TBI on the skeleton and its possible mechanisms.

## Posttraumatic Mortality and Morbidity

The clinical severity of TBI has been associated with an increased risk for mortality. Per the CDC, there are over 50,000 deaths in the U.S. annually and approximately 22% die within the first 5 years after suffering a TBI. TBI patients (mild, moderate, or severe) with brain edema were eight times as likely to die compared to TBI patients without edema and surprisingly, were five times as likely to die for mild TBI-related edema (4). Those who survive moderate-to-severe TBI and receive rehabilitation have a shorter life expectancy by 9 years (5).

A diffusion tensor imaging study of the corpus callosum found that those with mild TBI had significant white matter abnormalities up to 3 months post-injury (6). These studies suggest that mild TBI, previously considered harmless, could in fact lead to more TBI-related morbidity.

## Neurological Disorders and Neurodegenerative Diseases

Trauma-induced neurological and neurodegenerative sequalae are relatively common. An estimated 70% of adults in the United States have experienced a traumatic event in their lifetime, in which, approximately 20% go on to develop PTSD. These individuals are at an increased risk for psychological disorders (i.e., depression and anxiety), neurological disorders (i.e., epilepsy and sleep disorders), physical injuries, substance abuse, and fatigue, all of which can lead to poor decisions and actions and can manifest into physical symptoms.

Concussion, or mild TBI, accounts for approximately 90% of all brain traumas sustained (7). While many impairments resulting from a mild TBI tend to resolve within a few months, a subset of these individuals, in addition to moderate and severe TBI, exhibit gradual cognitive decline and motor deficits such as those that are characteristic in Alzheimer’s disease, Parkinson’s disease, and chronic traumatic encephalopathy (8).

## TBI Effects on the Skeletal System

Traumatic brain injury and its associated physiological processes have been found to lead to significant skeletal abnormalities that often transpire over time (Table 1). While the consequences and mechanisms of trauma to the head and the pathophysiological and neurochemical events that occur during the course of initial hours and days are being extensively investigated, little is known regarding the long-term consequence of TBI on remote organs that are under the influence of neural and neuroendocrine humoral outflow from the brain *via* the pituitary. Recently, the discovery of bone regulation by neural signals represents an emerging area of study that is identifying novel regulatory axes between the nervous system and bone cells. Based on the key role for cells of the hypothalamic nuclei in the neuro(endo)crine regulation of bone remodeling, it is predictable that injury to the brain will have a severe impact on the regulatory molecules that control skeletal growth/maintenance. The rest of this review will focus on the long-term consequences of TBI on the skeletal system and the potential mechanisms for TBI effects on bone.

**Table 1:** Examples of clinical studies showing relationship between TBI and bone-related abnormalities.

Bone abnormalities	Effects of TBI	Reference
Osteoporosis	21.4% had osteoporosis, 41.1% had osteopenia, and 27.7% were vitamin D deficient	Smith et al. (9)

Osteoporosis	19% had osteoporosis, 50% had osteopenia	Scarvell et al. (10)

Low BMD	Low BMD in the tibia and radius	Banham-Hall et al. (11)

Fracture	Increased risk of upper limb fracture with mTBI	Jodoin et al. (12)

Fracture	Accelerated fracture healing and enhanced callus formation	Yang et al. (13)

Fracture	Accelerated fracture healing, enhanced callus formation, and increased osteoblast proliferation	Cadosch et al. (14)

HO	11.4% developed HO after severe TBI	Simonsen et al. (15)

HO	22.5% developed HO after TBI	Citta-Pietrolungo et al. (16)

*TBI, traumatic brain injury; BMD, bone mineral density; mTBI, mild traumatic brain injury; HO, heterotopic ossification*.

### Heterotopic Ossification (HO)

Heterotopic ossification is defined as the formation of mature, lamellar bone, in non-osseous tissue, typically between the muscle and joint capsule. Although, there are rare hereditary disorders associated with HO, it is well established that HO is usually acquired following trauma to soft tissues, bone, and neurological damage. The incidence of ectopic bone formation is frequently seen in patients with TBI (17–22). This ectopic deposition of bone elicits symptoms including pain with a significant loss of range of motion (17, 23, 24).

Three subtypes of HO have been identified: genetic, traumatic, and neurogenic. Genetic HO occurs in individuals with inherited conditions such as fibrodysplasia ossificans progressive and progressive osseous heteroplasia. Traumatic HO occurs in response to injuries such as acetabular fractures, fractures and joint dislocations, blast injuries, burns, combat, amputee injuries, and muscle trauma (25–28). Neurogenic HO occurs in response to TBIs and spinal cord injuries (17, 29). Those with TBI alone go on to develop HO throughout the body, distant from the primary injury, in locations such as the hip, knee, elbow, or shoulder (30). Many returning from military combat develop HO from the combination of trauma and neurogenic injuries (18, 31–33). TBI occurrence with local tissue injury has been associated with the pathogenesis of HO and suggests that both systemic and local mechanisms may contribute to HO. Recently, serum collected from TBI patients promoted conversion of muscle cells into osteoblasts, suggesting that TBI may accelerate the formation of ectopic bone at the sites of injury (34).

The identification of cellular and molecular events leading to HO continue to remain elusive. Recent studies suggest that the activation of the bone morphogenic protein (BMP) signaling may play a significant role in the pathogenesis of HO based on the established role for BMPs to promote *de novo* bone formation in non-skeletal sites (35–38). Specifically, HO induction with BMP-2 led to the expression of osteoblast-specific transcription factors in the endonerium, suggesting that endoneurial progenitors are osteogenic precursors that contribute to HO (39). Further, osteoblasts isolated from HO bone (rodent and clinical models) exhibited increased osteogenic differentiation compared to osteoblasts derived from normal bone (40).

**Figure 1:**
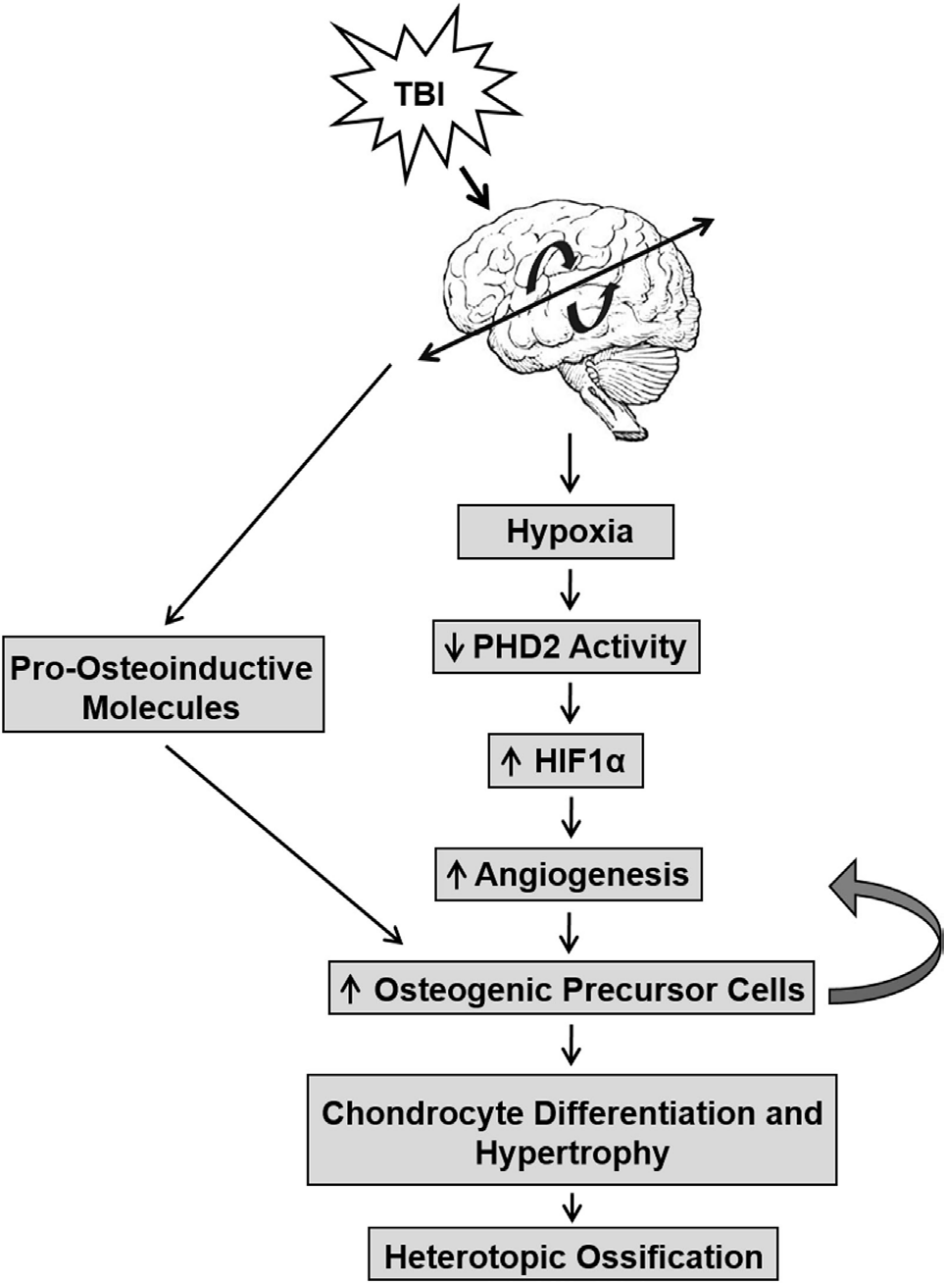
The interaction between traumatic brain injury (TBI) and hypoxic conditions that lead to the development of heterotopic ossification (HO). TBI induces a hypoxic environment in tissue that reduces PHD2 activity, which in turn, prevents the cleavage of hypoxia-inducible factor (HIF)1α and increases angiogenesis. This pathway increases osteogenic precursor cell activity, thereby promoting chondrocyte differentiation and hypertrophy in soft tissues and leading to bone formation in HO. TBI may also directly affect pro-osteoinductive molecules that promote increased osteogenic precursor cell activity.

Studies have also linked the release of pro-angiogenic factors to the onset of HO (Figure 1) (41–43). The vascularization of injured tissue represents an important step during the transition from hypertrophic cartilage to bone during the endochondral ossification of cartilage (44, 45). Neovascularization occurs within the injured tissue because of low oxygen tension that is caused by reduced activity of prolyl hydroxylase domain proteins (PHDs). The early response activity of PHD proteins are oxygen dependent and, under normoxia, hydrolyze hypoxia-inducible factor (HIF)-α subunits, resulting in their degradation and inactivity. Under hypoxia, PHD activity decreases due to reduced oxygen levels. Thus, the HIF-1α subunit avoids hydroxylation, accumulates in the nucleus, recruits HIF-1β, and together bind to penta-nucleotide hypoxia-responsive elements to promote transcription of key genes, such as VEGF, involved in the angiogenesis pathway (44, 46, 47). Hypoxic conditions also impact bone formation by stimulating oxygen-sensing factors that promote chondrocyte survival and differentiation (35, 48, 49). The increase in osteogenic precursor cells create a feedback loop to increase angiogenesis and exacerbate chondrocyte differentiation and hypertrophy. Conditional disruption of the *Phd2* gene in chondrocytes promoted endochondral ossification and dramatic increase in trabecular bone mass (50). It was found that loss of PHD2 in chondrocytes promoted chondrocyte differentiation and osteoblast formation and that this effect was in part mediated *via* upregulation of HIF1α signaling. The extent to which hypoxia signaling contributes to ectopic bone formation caused by brain injury remains to be elucidated.

### Fracture Healing

Fracture healing can be defined as the physiological repair of bone tissue, structure, and function after injury. Patients who have sustained severe TBI commonly demonstrate alterations in the healing process of bone. There is mounting evidence linking the association between TBI and enhanced osteogenesis postfracture. TBI in combination with fracture results in higher bone volume, higher mineral density (51), and accelerated healing with enhanced callus formation (14, 52–55).

The physiology of fracture healing involves both local and systemic factors that can be divided into three overlapping phases: inflammatory phase, followed by reparation, and finally remodeling. During the inflammatory phase, bleeding from the fracture and adjacent soft tissues result in the formation of hematoma at the injured site. The hemopoietic cells at the hematoma secrete cytokines and growth factors and attract osteoprogenitor and mesenchymal cells that result in the proliferation of osteoblasts and fibroblasts. Cytokines such as interleukin (IL)-6, produced by stromal/osteoblastic cells, enhance angiogenesis and osteoclastogenesis and regulate bone resorption and callus formation (56). During the reparative phase, a primary soft callus develops, followed by a medullary (hard) callus several weeks later. During the remodeling phase, the fracture site is reshaped by the interactions between osteoclasts resorbing bone and osteoblasts forming bone that strengthens the bone.

The existence of whether humoral osteogenic factors released post-TBI and/or direct nervous action guides the induction of enhanced fracture healing is still being debated. Several studies have detected humoral osteogenic factors in the serum of TBI patients (57) and in cells (14, 58). There is also evidence in the literature to suggest that a combination of signaling cascades involving humoral, neuronal, and local bone markers may play a significant role in fracture repair after trauma (59, 60). Cell proliferation was significantly increased in rat osteoblast cells treated with serum from TBI patients, suggesting the role of a circulating growth factor (either systemic or local) that promoted osteogenic activity (61). In another study, growth hormone (GH) levels continued to increase during enhanced osteogenesis with a gradual increase in IGF-1 during fracture healing in patients with combined TBI and fracture (62). Other growth factors have also been implicated in the process of fracture healing. Serum levels of epidermal growth factor and nerve growth factor gradually increase up to 14 days post-TBI and fracture combined injury compared to fracture or TBI groups alone (63).

**Figure 2:**
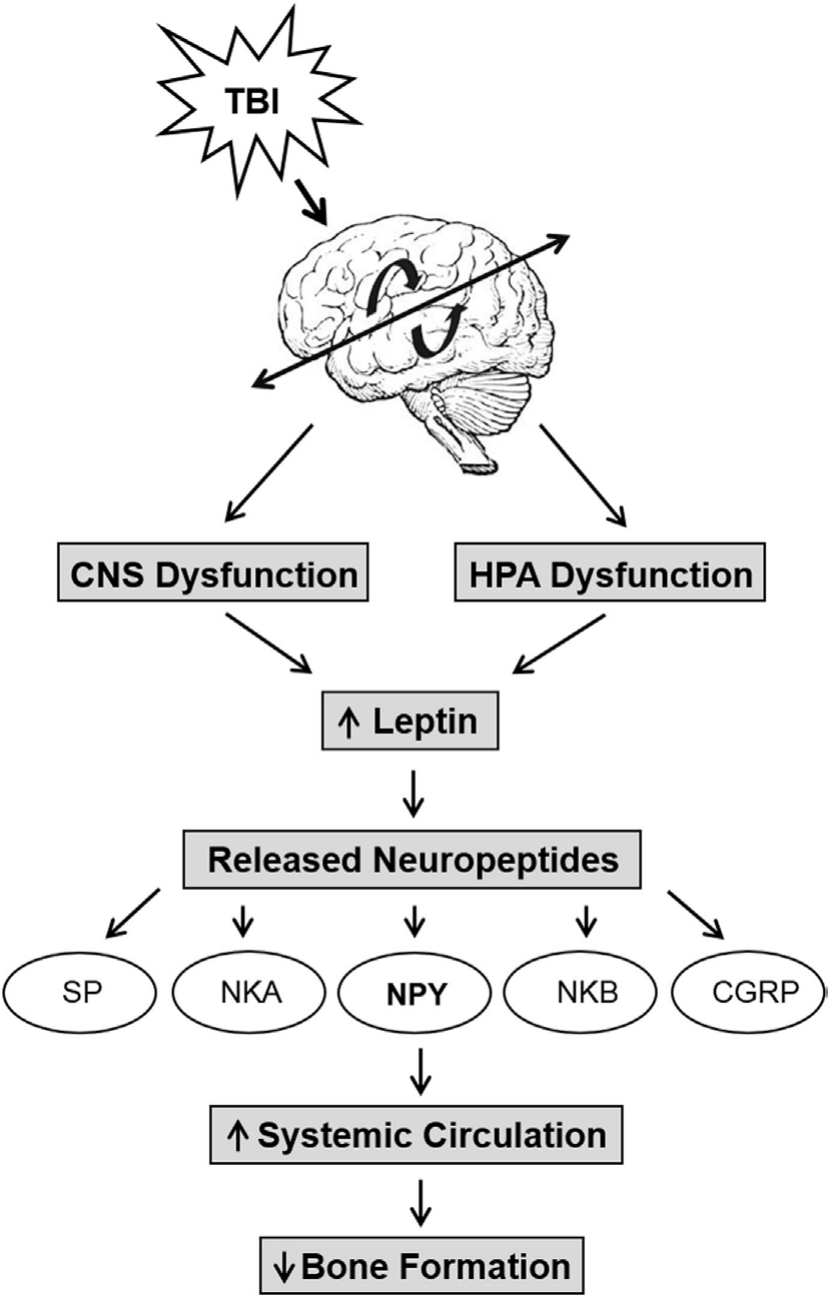
The relationship between trauma, neuropeptides, and decreased bone formation. Traumatic brain injury (TBI) induces central nervous system disruption and inflammation and causes an upregulation in leptin levels due to the compromised blood–brain barrier. TBI also causes hypothalamus–pituitary–adrenal axis (HPA) dysfunction that increases leptin and causes the release of neuropeptides such as substance P (SP), neurokinin A (NKA), neuropeptide Y (NPY), neurokinin B (NKB), and calcitonin gene-related peptide (CGRP). These neuropeptides propagate further inflammation that further increase systemic circulation of NPY and other neuropeptides that reduce bone formation *via* the leptin pathway.

The central and peripheral nervous systems play important roles in the regulation of bone with many factors influencing this densely innervated tissue in the body. One of these factors is leptin, and it has been hypothesized to be an integral part of fracture healing (Figure 2). Leptin is an adipocyte-derived hormone expressed in several tissues that primarily controls energy and food intake (64) and is involved in the regulation of insulin homeostasis, reproduction, immune function, and brain development (65). More recently, *in vivo* studies have shown elevated levels of GH and IGF-I in serum of TBI and fractured rabbits (66) that could be caused by increase in leptin levels presumably due to disruption of blood–brain barrier. In addition, higher percentage of leptin-positive cells in the callus have also been detected in combined TBI and fractured rats (67, 68). Further, studies using knockout mice suggest that changes in callus formation were not detected in leptin-deficient ob/ob mice that underwent TBI and osteonomy (69); however, peripheral injections of leptin significantly increased bone mass and osteoblastic activity in ob/ob mice (65, 70). Neuropeptide Y (NPY), a neurotransmitter with regulatory functions in bone homeostasis, is downstream of leptin signaling. NPY levels increase and promote osteogenic differentiation in patients with TBI and fractures (71). NPY and leptin share an inverse relationship, and recent studies suggest that NPY may mediate part of the skeletal phenotype in ob/ob mice (72, 73). Another factor is Semaphorin 3A, a secreted cytokine regulated by neural injury and is known to guide axonal and dendritic growth and neural migration. Recent studies have suggested that Semaphorin 3A can suppress bone resorption by binding to neurophilin-1 that in turn inhibits receptor activator of nuclear factor κB ligand-induced osteoclast differentiation and other pathways and synchronously stimulates osteoblasts and enhanced osteogenesis *via* canonical Wnt-signaling (74). In addition, stromal cell-derived factor-1, a chemokine protein, has been found to contribute to endochondral bone repair in TBI and fracture healing *via* increased expression surrounding tissues of fractured bone (75). Thus, while a number of signaling molecules have been implicated in the accelerated fracture healing caused by TBI, the mechanism by which brain injury influences these molecules and the extent to which the identified molecules interact to regulate fracture healing remain to be established.

### Low Bone Mass

Patients who have experienced TBI may have an increased risk of osteopenia and osteoporosis (9, 11), which are asymptomatic systemic skeletal diseases result in the micro-architectural deterioration of bone tissue leading to bone fragility and ultimately, fractures. Changes in bone are characterized by reduced bone mineral density (BMD) due from immobility and other metabolism-related factors. In animal models, TBI significantly reduces BMD in cortical bone (2, 76) and decreases in tibial trabecular bone mass (77) irrespective of mobility. In stroke patients, the highest rate of bone loss occurs within the first year (78), particularly, greatest loss of BMD in the paretic hip and upper limbs. Adults with TBI have been shown to have higher BMD loss in the femur and vitamin D deficiency (9). Several groups of stroke patients have also been shown to have deficiency in vitamin D levels (25-hydroxyvitamin D or 1,25-dihydroxyvitamin D) (79, 80). Although low vitamin D levels in stroke patients may be attributed to lack of exposure to sunlight following stroke and the hypoparathyroidism induced by immobilization hypercalcemia, it may also be that many stroke patients have insufficient levels of vitamin D prior to stroke (81).

Traumatic brain injury-induced skeletal changes seems to be dependent in part on the variations in parathyroid hormone (PTH) and vitamin D axis. TBI reduces PTH activity, thereby reducing 1,25-dihydroxyvitamin D production and activity. The resulting hypoparathyroidism and reduced serum calcium levels leads to increased bone resorption and reduced microstructural integrity (82), and, thereby, increasing risk for fractures (9, 83, 84).

## Mechanisms Detrimental Effects of TBI on Bone

### Inflammation

Activation of the inflammatory response in the brain occurs within a few seconds after trauma and with the permeation of the blood–brain barrier and activation of several injury cascades (85, 86). Injury to the central nervous system (CNS) results in an increase of blood products, tissue debris, prostaglandins, reactive oxygen specials, and nitrogen species. These factors, in turn, trigger an innate response of resident immune cells (i.e., macrophages, mast cells, granulocytes, dendritic cells, and natural killer cells) through the activation of microglia and astrocytes, increased migration and recruitment of leukocytes, and the release of inflammatory mediators such as cytokines and chemokines (pro- and/or anti-inflammatory) and results in local and systemic immune responses (87, 88). Systemic inflammation shifts toward an adaptive immune response and to the chronic stages post-TBI (days, weeks, months, years), which may, in fact, exacerbate the onset of skeletal deterioration. For example, increased systemic IL-6 and IL-11 may directly stimulate osteoclastic activity or act *via* osteoblast lineage cells that increase osteoclast formation through receptor activator of nuclear factor kappa-B ligand (89). Similarly, increased levels of systemic IL-18 has been associated with poor clinical outcome in TBI patients (90).

### Neuropeptides

Trauma also leads to neurogenic inflammation, where the activation of sensory unmyelinated neurons by noxious stimuli (i.e., TBI) causes the simultaneous release of neuropeptides such as substance P (SP), neurokinin A, neuropeptide Y (NPY), neurokinin B, and calcitonin gene-related peptide as shown in Figure 2. The release of peptides results in vasodilation, increased vascular and blood–brain barrier permeability (91). The compromised blood–brain barrier causes an influx of plasma proteins that enhances the inflammatory response, as well as creating a positive feedback with neurogenic inflammation to propagate further inflammation with the release of pro-inflammatory mediators, oxidative factors, etc., which causes further neural damage. In addition, the disruption of the hypothalamus may increase the circulation of peripheral NPY and reduce bone formation *via* leptin (72, 73).

### Hypothalamus–Pituitary–Adrenal Axis (HPA)

Together with the central nervous and circulatory systems, the HPA axis, also the endocrine system, is critical for the integration and coordination for many bodily functions such as stress reactions, digestion, immune system, mood and emotions, sexuality, etc. The HPA axis acts as a complex integration and feedback network between three endocrine glands: hypothalamus, pituitary gland, and the adrenal gland. The hypothalamus synthesizes and secretes vasopressin and corticotropin-releasing hormone, which in turn stimulates the secretion of adrenocorticotropic hormone in the pituitary gland and further stimulates the production of glucocorticoid hormones. The glucocorticoid hormones in turn act back on the hypothalamus in a negative feedback loop, secreting hormones as necessary for function. In healthy individuals, HPA signaling maintains homeostasis. In response to brain injury, the function of the HPA axis is disrupted, causing a reduction in the production of many endocrine factors (hypopituitarism; Figure 1) (92, 93). It has been reported that 70% of patients with TBI have hypothalamic–pituitary dysfunction (94). For example, a 12-month prospective study found that the rate of hypopituitarism in post-TBI patients was reduced by 33 and 23% at 3 and 12 months, respectively (95). Another clinical study also reported anterior pituitary dysfunction in 56 and 36% of TBI patients at 3 and 12 months post injury, respectively (96). These studies indicate that HPA dysfunction is a key consequence of TBI.

**Figure 3:**
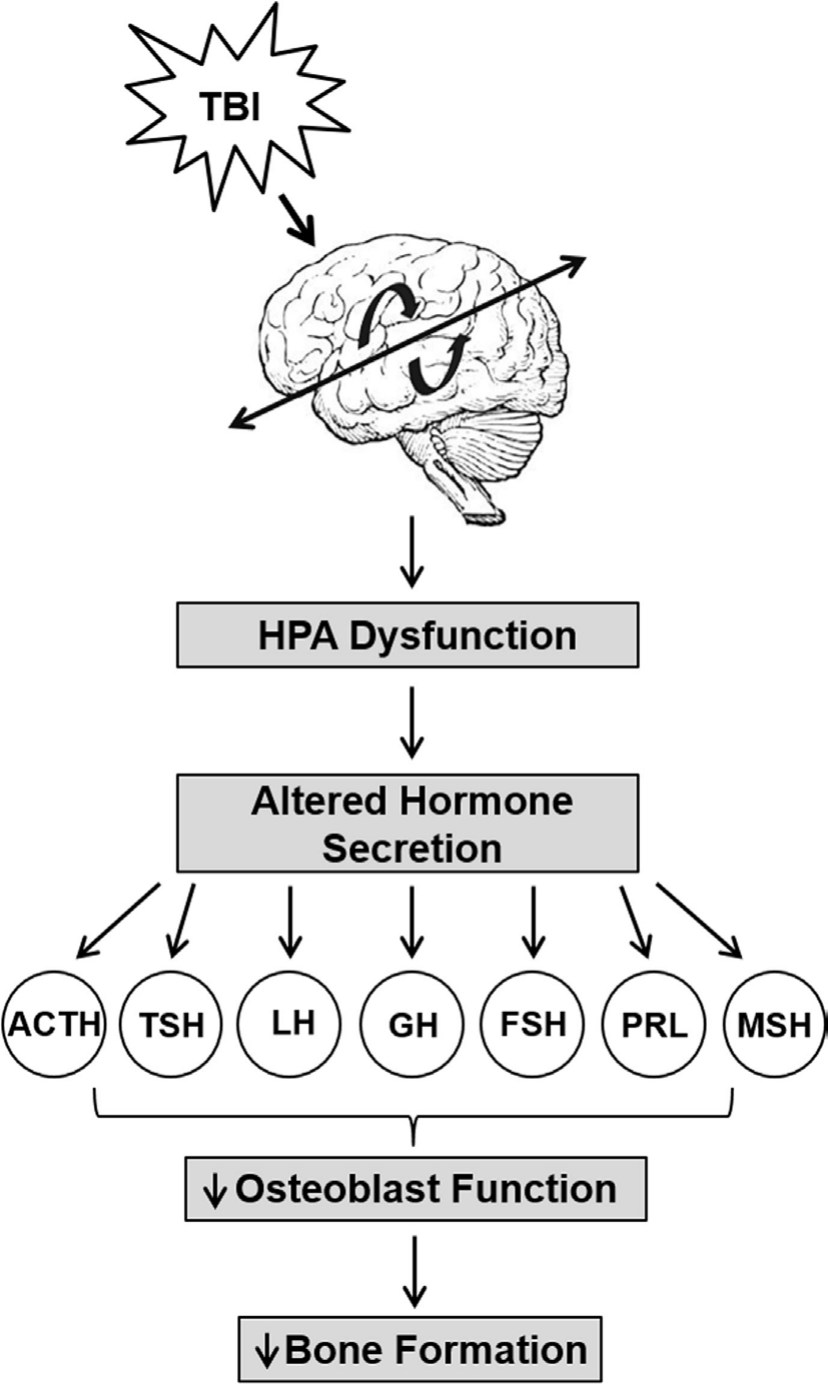
Trauma-induced hypothalamus–pituitary–adrenal axis (HPA) dysfunction lead to reduced bone formation. Traumatic brain injury causes significant HPA dysfunction that leads to increased levels of adrenocorticotropin (ACTH), prolactin (PRL), and growth hormone (GH), but decreased or unchanged levels in luteinizing hormone (LH), follicle-stimulating hormone (FSH), PRL, melanocyte-stimulating hormone (MSH), and thyrotropin (TSH) levels. The altered secretion of hormones impact osteoblast function and impair bone formation.

Hypopituitarism is the most common manifestation of the HPA axis disruption *via* TBI that causes hormonal deficiencies (Figure 3) (97–101). Individuals suffering from TBI are at the risk of developing long-term negative consequences on the skeleton *via* deficiencies in GH, gonadotrophin, and thyroid-stimulating hormone (TSH), all of which significantly influence bone metabolism. For example, GH is known to have anabolic and catabolic mechanistic pathways that may be injury dependent in bone. GH interacts with insulin-like growth factor-1 and both contribute to the regulation of bone mass through the stimulation of osteoblast activity (102, 103). Deficiency in GH created a catabolic effect that increases skeletal fragility and low bone mass that leads to higher fracture risk (104) and delayed fracture healing (105). However, GH creates an anabolic effect by stimulating accelerated fracture healing (106, 107). TSH directly inhibits osteoclast skeletal remodeling and osteoblast bone formation (108) and lower levels of TSH significantly increases bone resorption and reduces bone osteogenesis (109). Pituitary hormone disturbances are frequently found after TBI and lead to changes in bone mineralization (110). These hypothalamic–pituitary changes may be exacerbated in repetitive trauma. We have found that mild repetitive TBI led to significant loss skeletal mass (2), and more recently, we have shown that these negative effects on bone microarchitecture and mechanical properties were mediated by osteoblast function *via* reduced endocrine IGF-1 actions (111). Recent studies also demonstrate an important role for central control of bone mass involving leptins and neuropeptides (112, 113). Thus, the hormonal disruption of the HPA leads to many detrimental skeletal effects.

## Clinical Recommendations

The physical presentation of bone metabolism abnormalities post-TBI may not be detected until considerable skeletal damage has already occurred. We recommend the assessment of serum from TBI patients for levels of biochemical markers of bone metabolism (i.e., bone-specific alkaline phosphatase, osteocalcin, *N*-terminal propeptide of type I procollagen, *N*-terminal telopeptide of type I collagen, and C-terminal telopeptide of type I collagen) and GH deficiency (GH, insulin-like growth factor-I, IGF binding protein-3, and acid labile subunit) (60) as the first line of preventative treatment. Patients with increased serum markers of bone turnover and GH deficiency would benefit from DXA imaging to monitor skeletal deficits across time. Changes in serum levels of bone markers can be used to further tailor pharmacological interventions toward promoting bone formation (PTH, sclerostin antibody) or inhibiting bone resorption (bisphosphonates). Thus, a multifaceted treatment approach is necessary for the detection and prevention of skeletal deficit in TBI patients.

## Conclusion

Traumatic brain injury leads to significant functional impairments and structural alterations that can lead to permanent long-term changes in TBI survivors. These changes include sleep disturbances, impaired movement, cognitive dysfunction, and neurodegenerative diseases. Alterations in the CNS, as well as the disruption of the HPA axis alter skeletal remodeling processes and enhance resorption in sites distal to the site of injury, ultimately leading to low bone mass and skeletal fragility. Interestingly, TBI stimulates osteoblasts and enhanced osteogenesis in fractured healing bones. We conclude that TBI is a complex multifaceted process that leads to acute and long-term skeletal consequences that directly impacts bone integrity and function in TBI survivors.

## Future Perspectives

Although several studies have revealed key mechanisms in the pathogenesis of TBI-induced bone alterations, particularly in the release of humoral and osteogenic factors, many mechanistic pathways remain to be explored to fully understand the processes. Further studies are needed to investigate the consequences of moderate to severe TBI and whether the mechanistic pathways leading to skeletal alterations are similar or different. These investigations would lead to pharmacological approaches targeting pathways simultaneously may aid in preventing adverse skeletal changes.

## Author Contributions

NB, CK, and SM wrote the manuscript. NB prepared tables and figures. SM revised and approved the final manuscript.

## Conflict of Interest Statement

The authors declare that the research was conducted in the absence of any commercial or financial relationships that could be construed as a potential conflict of interest.
